# Supplementary material for: Investigating the dynamics of microbial consortia in spatially structured environments
Source: Nat Commun. 2020 May 15;11:2418. doi: 10.1038/s41467-020-16200-0 (PMC7228966; doi:10.1038/s41467-020-16200-0)
Supplement: Supplementary file 5 — Description of Additional Supplementary Files [file 41467_2020_16200_MOESM5_ESM.pdf]

Title: Supplementary Movie 1
Description: Gene expression dynamics of the sender-receiver consortium to a forced oscillatory input. The spatially separated sender-receiver E. coli consortium communicates unidirectionally by diffusion of a quorum sensing molecule (AHL). The sender strain (represented by green) expresses the arabinose-inducible synthetase LuxI transcriptionally fused to GFP, which produces AHL. AHL diffuses through the interaction channels and into the receiver cells (represented by red) leading to induction of RFP driven by the receptor LuxR. The cells are exposed to an forced oscillatory arabinose input with a period of two hours that starts at the denoted media switch (Experiment 3, Table 1). The amplitude of the oscillations in RFP intensities at steady state decrease with the distance separating the sender and receiver strains.

Title: Supplementary Movie 2
Description: Oscillatory dynamics of a distributed genetic oscillator in defined spatial arrangements. Spatially separated strains harboring a distributed genetic oscillator grow in the MISTiC microfluidic device. The activator strain (CFP) highlighted in blue activates both itself (positive feedback) and the repressor strain (positive inter-strain interaction) while the repressor strain (YFP) represented by yellow represses both itself (negative feedback) and the activator strain (negative inter-strain interaction). The oscillatory dynamic are induced by IPTG at the denoted media switch. (Experiment 5, Table 1). The oscillations in CFP (activator) are more robust to variations in spatial separation and exhibit lower variability compared to YFP (repressor).

Title: Supplementary Movie 3
Description: Growth dynamics of a mixed E. coli amino-acid auxotroph consortium in MISTiC. E. coli auxotroph strains ΔmetA and ΔpheA grow as a mixed community in growth chambers within MISTiC (Experiment 8, Table 1). Raw phase contrast footage is augmented with fluorescence data to display ΔmetA cells as green and ΔpheA cells as red. The cell segmentation (phase contrast) for each chamber is shown on the left. The community is grown in media containing M and F until the denoted media switch, at which point these amino acids were removed from the environment.
