# Supplementary material for: Investigating the dynamics of microbial consortia in spatially structured environments
Source: Nat Commun. 2020 May 15;11:2418. doi: 10.1038/s41467-020-16200-0 (PMC7228966; doi:10.1038/s41467-020-16200-0)
Supplement: Supplementary file 6 — Supplementary Information [file 41467_2020_16200_MOESM6_ESM.pdf]

Supplementary Information

Investigating the dynamics of microbial consortia in spatially structured environments

Gupta et al.

1 Supplementary Methods

1.1 Modeling diffusion through the interaction channel

We assumed that diffusion through the MISTiC interaction channels could be modeled as a one-dimensional process by discretizing space in the interaction channels into 1 micron regions. The chamber connected to the main channel with 10 μM fluorescein is the 'source' chamber, x_1, through which the dye enters the interaction channel and diffuses across at rate D_1. The concentration in the source chamber remains constant where

$$\dot{x_1} = 0. \tag{1}$$

We assumed that fluorescein degraded at a linear rate due to absorption by PDMS or photobleaching. The concentration of fluorescein in each region of the interaction channel is

$$\dot{x_l} = D_1(x_{l-1} + x_{l+1} - 2x_l) - \gamma_F x_l \text{ for } l = 2, 3, ..., L-1. \tag{2}$$

The 'sink' chamber, x_L is on the opposite side of the interaction channel, through which fluorescein dilutes into the other main channel at a rate D_2. Fluorescein concentrations in the sink chamber are given by

$$\dot{x_L} = D_1(x_{L-1} - x_L) - D_2 x_L - \gamma_F x_L. \tag{3}$$

The model parameters (Table S1) were fit to the fluorescence data within the interaction channel using a nonlinear programming solver (fmincon, MATLAB). For $\gamma_F = 0$, the model exhibits linear chemical gradients that do not mirror the shape of the experimentally measured gradients (**Fig. S1**).

Parameter	Description	Value
D_1	Diffusion coefficient of fluorescein across the interaction channel	2.0016 x 10^5 time^{-1}
γ_F	Degradation rate of fluorescein	7.4466 time^{-1}
F_c	Fluorescein concentration in source chamber	0.9135 A.U.
D_2	Diffusion coefficient of fluorescein into main channel	1227.8 time^{-1}

Supplementary Table 1: Estimated parameters for the fluorescein experiment.

1.2 Dynamic computational model of inter-strain communication across spatial distance

An ordinary differential equation model was developed to study quorum-sensing inter-strain communication across distance. Reflecting our diffusion model, we assumed that diffusion could be modeled as a one-dimensional process by discretizing space in the interaction channels into 1 micron regions. Dynamical delays were used to model sequential biochemical reactions, syringe activation, and transport of media from the inlets into the growth chambers. The model species are listed in Table S2.
The following equations account for the time delay due to arabinose transport and diffusion into the device:

$$\dot{w_1} = a_1(ara - w_1), \tag{4}$$

$$\dot{w_i} = a_1(w_{i-1} - w_i) \text{ for } i = 2 : N_1 - 1, \tag{5}$$

$$\dot{w_{N_1}} = D_0(w_{N_1-1} - w_{N_1}). \tag{6}$$

The species w_{N_1} represents the concentration of arabinose in the sender growth channel. The species GFP_m is modeled as

Species	Description
$w_i, i = 1, 2, ..., N_1$	Arabinose concentration in different regions of the device
GFP_m	GFP mRNA
$v_i, i = 1, ..., N_2$	GFP protein intermediates
v_{N_2}, GFP_p	Mature GFP protein
$LuxI_m$	LuxI mRNA transcript
$u_i, i = 1, ..., N_3$	LuxI protein intermediates
$u_{N_3}, LuxI_p$	LuxI protein
$x_i, i = 1, 2, ..., L-1$	AHL concentration in each region of the interaction channel
x_L, AHL	AHL concentration within the receiver chamber
$LuxRAHL$	Activated complex of $LuxR$ bound to AHL
RFP_m	RFP mRNA
$z_i, i = 1, ..., N_4$	RFP protein intermediates
z_{N_4}, RFP_p	RFP protein

Supplementary Table 2: Species descriptions for inter-strain communication model.

$$\dot{GFP_m} = \alpha_{LuxI} \frac{w_{N_1}^{n_{LuxI}}}{K_{LuxI}^{n_{LuxI}} + w_{N_1}^{n_{LuxI}}} - \gamma_{GFP} GFP_m. \tag{7}$$

To account for time delays associated sequential assembly and maturation of GFP, the following delay equations were used:

$$\dot{v_1} = a_2(GFP_m - v_1), \tag{8}$$

$$\dot{v_j} = a_2(v_{j-1} - v_j) \text{ for } j = 2 : N_2. \tag{9}$$

The species v_{N_2} represents concentration of mature GFP protein in the sender strain. The concentration of $LuxI_m$ was modeled as

$$\dot{LuxI_m} = \alpha_{LuxI} \frac{w_{N_1}^{n_{LuxI}}}{K_{LuxI}^{n_{LuxI}} + w_{N_1}^{n_{LuxI}}} - \gamma_{LuxI} LuxI_m. \tag{10}$$

Similarly to GFP, we represent delays in translation of LuxI protein using the following equations:

$$\dot{u_1} = a_3(LuxI_m - u_1), \tag{11}$$

$$\dot{u_k} = a_3(u_{k-1} - u_k) \text{ for } k = 2 : N_3. \tag{12}$$

The species u_{N_3} denotes the concentration of $LuxI_p$ in the sender strain. LuxI synthesizes AHL at a rate p_{AHL}, which diffuses into the interaction channel with a diffusion coefficient of D_1 and into the main channel with a diffusion coefficient of D_2. D_2 is significantly lower than D_1 since the diffusion rate through the cells is slower than the diffusion through the media. The equation representing AHL in the sender growth chamber is

$$\dot{x_1} = D_1(x_2 - x_1) - D_2 x_1 + p_{AHL} u_{N_3} - \gamma_{AHL} x_1. \tag{13}$$

The interaction chamber of length L is discretized into approximately 1 μm units, with x_2 and x_{L-1} representing the region closest to the sender growth chamber or receiver growth chamber, respectively. The concentration of AHL in each region of the interaction channel is

$$\dot{x_l} = D_1(x_{l-1} + x_{l+1} - 2x_l) - \gamma_{AHL} x_l \text{ for } l = 2, 3, ..., L-1. \tag{14}$$

The concentration of AHL in the receiver growth chamber is a balance between diffusion from the interaction channel and diffusion into the main channel and is modeled as

$$\dot{x_L} = D_1(x_{L-1} - x_L) - D_2 x_L - \gamma_{AHL} x_L. \tag{15}$$

In the receiver strain, we assume that the binding of AHL to LuxR is significantly faster than the timescales of transcription and translation. Therefore, based on the quasi-steady-state assumption, the concentration of activated LuxR is modeled as an algebraic equation

$$LuxRAHL = LuxR_{tot} \frac{x_L}{K_{LuxR} + x_L}. \tag{16}$$

The species $LuxRAHL$ activates the expression of RFP_m using the following equation

$$\dot{RFP_m} = \alpha_{RFP} \frac{LuxRAHL^{n_{RFP}}}{K_{RFP}^{n_{RFP}} + LuxRAHL^{n_{RFP}}} - \gamma_{RFP} RFP_m. \tag{17}$$

We model time delays associated with translation and maturation of RFP_p using the following equation

$$\dot{z_1} = a_4(RFP_m - z_1), \tag{18}$$

$$\dot{z_m} = a_4(z_{m-1} - z_m) \text{ for } m = 2 : N_4. \tag{19}$$

The species z_{N_4} denotes the concentration of mature RFP_p in the receiver strain.

1.3 Model of spatially separated amino-acid cross-feeding in microbial consortia

We constructed an ordinary differential equation model to study the effects of separation distance on amino acid cross-feeding dependent growth rates of strains in microbial communities. Similar to the quorum-sensing model, we assume that diffusion is modeled as a one-dimensional process by discretizing space in the interaction channels into one-micron regions. The diffusion coefficients of methionine (M) and phenylalanine (F) were set to the values of the inferred diffusion coefficients in the quorum-sensing model since these small molecules have similar molecular weights ($149 - 213\ g\ mol^{-1}$).

1.3.1 Intracellular transport of amino acids from the extracellular environment

We compute the intracellular concentrations of M and F based on the extracellular concentration using a previous method (Perez AM et al. *Molecular Systems Biology* 2017). The intracellular concentration of M in the $\Delta metA$ strain is given by

$$[M]_{\Delta metA} = U_M \frac{[M]_{ext}}{\kappa_M + [M]_{ext}} + [M]_{ext}, \tag{20}$$

where

$$U_M = \frac{V_{max,M} P_{tot}}{D_c} \quad \text{and} \quad \kappa_M = \frac{V_{max,M}}{k_M}. \tag{21}$$

Here, $[M]_{ext}$ is the concentration of extracellular methionine, D_c is the diffusion coefficient of amino acid across the outer membrane, $V_{max,M}$ is the uptake rate of M, P_{tot} is the concentration of membrane transporters, and k_M is the binding affinity of M to the transporter. Similarly, the concentration of F in the $\Delta pheA$ strain is

$$[F]_{\Delta pheA} = U_F \frac{[F]_{ext}}{\kappa_F + [F]_{ext}} + [F]_{ext}, \tag{22}$$

where

$$U_F = \frac{V_{max,F} P_{tot}}{D_c} \quad \text{and} \quad \kappa_F = \frac{V_{max,F}}{k_F}. \tag{23}$$

Parameter	Description	Value
ara	Arabinose concentration	0.0619 A.U.
a_1	Time-varying rate coefficient	1.3187 min^{-1}
N_1	Number of delay equations (arabinose)	6
D_0	Diffusion coefficient into growth chamber	0.057 min^{-1}
α_{LuxI}	$LuxI_m$ maximum transcription rate	264.53 A.U. min^{-1}
n_{LuxI}	LuxI Hill coefficient	2.02
K_{LuxI}	$LuxI_m$ half-maximum concentration	33.42 A.U.
γ_{LuxI}	$LuxI_m$ degradation rate	0.046 min^{-1}
N_3	Number of delay equations for LuxI translation & maturation	6
γ_{GFP}	GFP mRNA degradation rate	0.022 min^{-1}
a_2	Time-varying rate coefficient	1.2690 min^{-1}
N_2	Number of delay equations for GFP translation and maturation	15
a_3	Time-varying rate coefficient	0.5607 min^{-1}
D_1	Diffusion coefficient for AHL diffusion into interaction channel	7677.8 min^{-1}
D_2	Diffusion coefficient for AHL diffusion into main channels	251.0 min^{-1}
p_{AHL}	AHL production rate	564.5 min^{-1}
γ_{AHL}	AHL degradation rate	0.086 min^{-1}
$LuxR_{tot}$	Total concentration of LuxR protein	9.5 A.U.
K_{LuxR}	Half-maximum concentration for $LuxRAHL$	54.2 A.U.
α_{RFP}	Maximum transcription rate for RFP_m	184 A.U. min^{-1}
n_{RFP}	Hill coefficient for RFP transcription	1.04
K_{RFP}	Half-maximum concentration for RFP_m	101.3 A.U.
γ_{RFP}	RFP_m degradation rate	0.037 min^{-1}
a_4	Time-varying rate coefficient	0.9195 min^{-1}
N_4	Number of delay equations for RFP translation/maturation	4

Supplementary Table 3: Parameter values for quorum-sensing model. Time delays can be computed as the ratio of the number of delay equations to the time-varying rate coefficient. This yields 6.0 min for arabinose transport from the inlets into the sender growth chambers, 11.8 min for GFP translation and maturation and 4.4 min for RFP translation and maturation.

Species	Description
$[M]_{\Delta metA}$	Intracellular [M] in $\Delta metA$
$[F]_{\Delta pheA}$	Intracellular [F] in $\Delta pheA$
$x_{1,...,L}$, $[M]_{ext}$	Extracellular [M] in different regions of the interaction channel or growth chambers
$y_{1,...,L}$, $[F]_{ext}$	Extracellular [F] in different regions of the interaction channel or growth chambers

Supplementary Table 4: Species descriptions for auxotroph model.

Here, $[F]_{ext}$ denotes the extracellular F concentration, $V_{max,F}$ is the uptake rate of M, and k_F is the binding affinity of F to its transporter. We assume that D_c and P_{tot} are equivalent for both strains. The kinetic parameters $V_{max,M}$, $V_{max,F}$, κ_M, and κ_F were derived from Piperno JR and Oxender DL *Journal of Biological Chemistry* 1968 (**Table S5**).

1.3.2 Amino acid limited growth rates of auxotroph strains

We assume that growth of each strain is limited by the concentration of the amino acid that this strain is deficient to produce. Therefore, the instantaneous normalized growth rate of each strain is a function of the intracellular concentration of the amino acid. We assume a different non-zero basal growth rate for $\Delta metA$ and $\Delta pheA$ in the absence of the rescuing amino acid denoted by r_M and r_F, respectively. The normalized

growth rates for $\Delta metA$ and $\Delta pheA$ are given by the equations

$$GR_{\Delta metA} = (1 - r_M)\frac{[M]_{\Delta metA}^{b_M}}{M_{EC50}^{b_M} + [M]_{\Delta metA}^{b_M}} + r_M, \tag{24}$$

$$GR_{\Delta pheA} = (1 - r_F)\frac{[F]_{\Delta metA}^{b_F}}{F_{EC50}^{b_F} + [F]_{\Delta pheA}^{b_F}} + r_F. \tag{25}$$

The parameters M_{EC50} and F_{EC50} represent the concentrations of M and F required by $\Delta metA$ and $\Delta pheA$, respectively, to achieve a half-maximal growth rate. The parameters b_M and b_F are Hill coefficients that map the concentration of the amino acid to the growth rate of each strain.

1.3.3 Production and diffusion of amino acids

We model the production and diffusion of the amino acids M and F between growth chambers. The $\Delta pheA$ and $\Delta metA$ strains are located in the left and right growth chambers and produce M and F that diffuse through the interaction channel to the opposite end of the channel. The strains $\Delta pheA$ or $\Delta metA$ release and consume M or F. We assume that the amino acid consumption rate cannot be larger than its production rate. The maximum net production of M and F is represented by $\alpha_{net,M}$ and $\alpha_{net,F}$ and is defined as the maximum difference between the production rate minus the consumption rate of each amino acid. We model the net production, degradation, and diffusion of M and F. Let $x_i = [M]$ for $i = 1, ..., L$ denote the concentration of M in the ith unit of the channel.

$$\dot{x}_1 = D_1(x_2 - x_1) - D_2 x_1 - \gamma_M x_1 + \alpha_M \frac{(GR_{\Delta pheA}/U_F)^{c_M}}{(GR_{\Delta pheA}/U_F)^{c_M} + 1} \tag{26}$$

$$\dot{x}_i = D_1(x_{i-1} + x_{i+1} - 2x_i) - \gamma_M x_i \qquad \text{for } i = 2, 3, \ldots, L-1 \tag{27}$$

$$\dot{x}_L = D_1(x_{L-1} - x_L) - D_2 x_L - \gamma_M x_L \tag{28}$$

$$\tag{29}$$

Similarly, let $y_i = [F]$ for $i = 1, ..., L$ denote the concentration of F in the ith unit of the channel.

$$\dot{y}_1 = D(y_2 - y_1) - D_2 y_1 - \gamma_F y_1 \tag{30}$$

$$\dot{y}_k = D(y_{i-1} + y_{i+1} - 2y_i) - \gamma_F y_i \qquad \text{for } i = 2, 3, \ldots, L-1 \tag{31}$$

$$\dot{y}_L = D(y_{L-1} - y_L) - D_2 y_L - \gamma_F y_L + \alpha_F \frac{1}{((GR_{\Delta metA}/U_M)^{c_F} + 1)} \tag{32}$$

$$\tag{33}$$

Note that $[M]_{ext} = x_L$ and $[F]_{ext} = y_1$. The parameters γ_M and γ_F denote degradation rates of M and F, α_M and α_F are maximum production rates of the amino acids, and c_M and c_F are Hill coefficients for amino acid production. Consistent with our metabolite measurements, the net production of M is proportional to the growth rate of $\Delta pheA$, whereas the net production of F is inversely proportional to the growth rate of $\Delta metA$.

Parameter	Description	Value
α_M	Maximum production of M by $\Delta pheA$	0.045 mM min^{-1}
α_F	Maximum production of F by $\Delta metA$	0.55 mM min^{-1}
c_M	Hill coefficient of M production	1.35
c_F	Hill coefficient of F production	1.63
U_M	Maximum uptake rate of M by $\Delta metA$	0.022 min^{-1}
U_F	Maximum uptake rate of F by $\Delta pheA$	4.79 min^{-1}
M_{EC50}	[M] that enables half-maximum growth of $\Delta metA$	0.35 mM
F_{EC50}	[F] that enables half-maximum growth of $\Delta pheA$	0.12 mM
r_M	Basal growth rate of $\Delta metA$ in absence of M	0.073 min^{-1}
r_F	Basal growth rate of $\Delta pheA$ in absence of F	0.21 min^{-1}
γ_M	Degradation rate of methionine	0.073 min^{-1}
γ_F	Degradation rate of phenylalanine	0.044 min^{-1}
b_M	Hill coefficient of $\Delta metA$ growth	1.81
b_F	Hill coefficient of $\Delta pheA$ growth	4.06
D_1^*	Diffusion coefficient through interaction channel	7677.8 μm min^{-1}
D_2^*	Diffusion coefficient from growth chambers to main channels	251.0 μm min^{-1}
$V_{max,M}^+$	Maximum uptake rate of M	0.39
$V_{max,F}^+$	Maximum uptake rate of F	0.75
κ_M^+	Ratio of uptake rate to transporter binding affinity of M	0.0023 mM
κ_F^+	Ratio of uptake rate to transporter binding affinity of F	0.00072 mM
P_{tot}/D_c	Diffusion coefficient of amino acid across cell membranes	1

Supplementary Table 5: Parameter values for auxotroph model. The symbols * and + indicates parameters from the quorum-sensing model or previous literature (Piperno JR and Oxender DL *Journal of Biological Chemistry* 1968), respectively.

2 Supplementary Figures

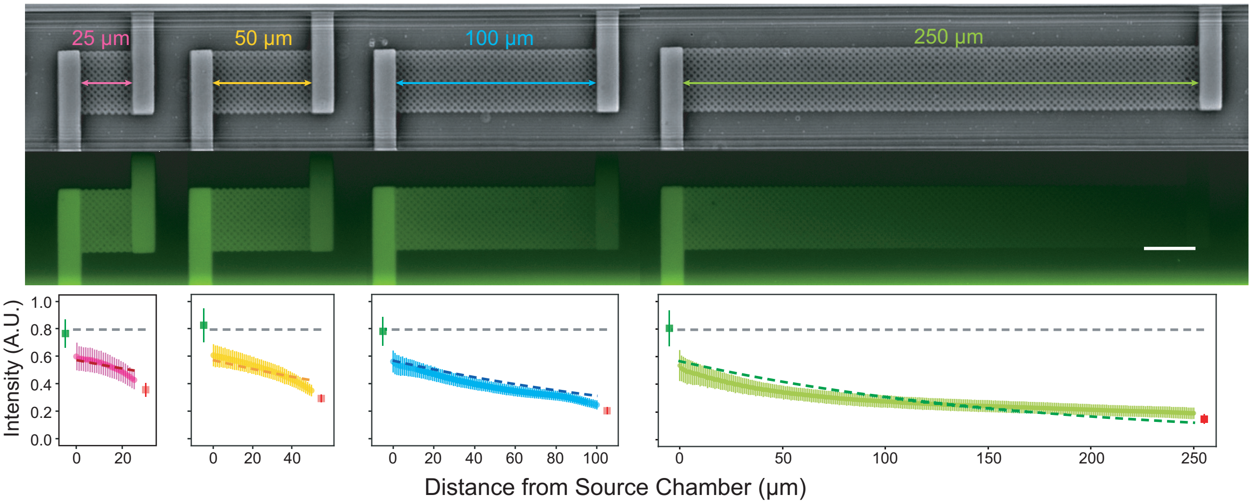

Supplementary Figure 1: Fluorescein dye demonstrates chemical gradients established within MISTiC. Top: Phase contrast and fluorescence microscopy images of the microfluidic device. Fluorescein and water were continuously administered through separate main channels. The scale bar represents 25 μm. **Bottom:** fluorescence intensity in the growth chambers and interaction channels as a function of distance from the source chamber. Green and red squares represent the average intensity within three independent replicates of source and sink chambers, respectively. The difference in the average fluorescence intensity between the 25 μm and 250 μm source channel was not statistically significant (P = 0.7539) based on a two-sided t-test. The difference in the average fluorescence intensity between the 25 μm and 250 μm sink channels was statistically significant (P = 0.0083) based on a two-sided t-test. The grey dashed line represents the mean fluorescence intensity in the source chambers across the different interaction channel lengths. Dashed lines represent the model fit to the data. Data points represent average fluorescein concentration in 1 μm regions of the interaction channels. Error bars represent one standard deviation from the mean of three independent replicates in different regions of the device. This experiment was repeated twice using slightly different protocols, with similar fluorescein gradients observed across the interaction channels in both experiments.

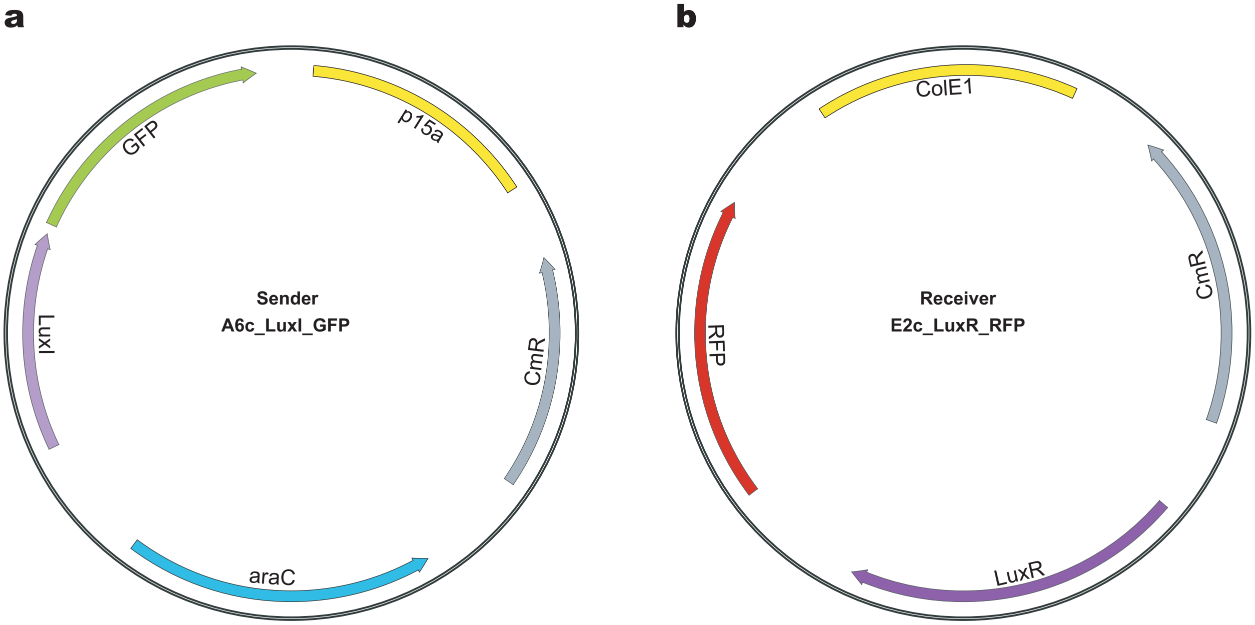

Supplementary Figure 2: Plasmid maps for sender-receiver quorum sensing experiments. (a) Plasmid map for A6c_LuxI_GFP, which was transformed into the sender *E. coli* strain BW27783 (**Methods**). The arabinose-inducible P_{BAD} promoter activates transcription of the operon containing the synthetase LuxI and fluorescent reporter GFP. **(b)** Plasmid map for E2c_LuxR_RFP that was transformed into the receiver *E. coli* strain MG1655z1 (**Methods**). The key features include the receptor LuxR regulated by the aTc-inducible P_{TET} promoter and the fluorescent reporter RFP driven by a P_{LuxI} promoter.

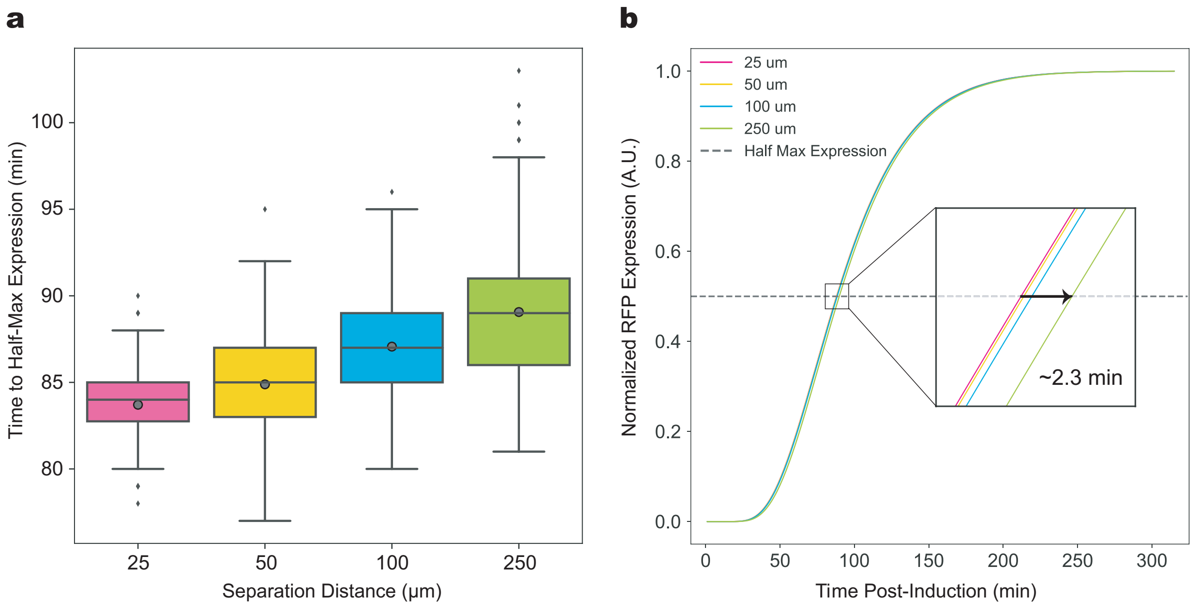

Supplementary Figure 3: Characterization of time delays in quorum-sensing chemical signal communication. **(a)** Experimentally measured time delays for the quorum-sensing synthetic community as a function of the distance separating the sender and receiver strains (Experiment 1, **Table 1**). The distributions were computed by bootstrapping the RFP fluorescence as a function of time (1000 resampled curves) (**Methods**). For each resampled curve, the time delay was calculated by normalizing the curve between 0 and 1 and determining the time to reach 0.5. Lines and circles within the boxes represent the median and mean of the distributions, respectively. Upper and lower box edges represent upper and lower quartiles, respectively. Upper and lower whiskers represent the 95th and 5th confidence intervals, respectively. **(b)** Normalized model simulations of RFP_p over time for different distances from the sender strain. Increasing the distance from 25 μm to 250 μm resulted in a 2.3 min time delay computed as the time to reach the half-maximum RFP_p concentration (dashed line).

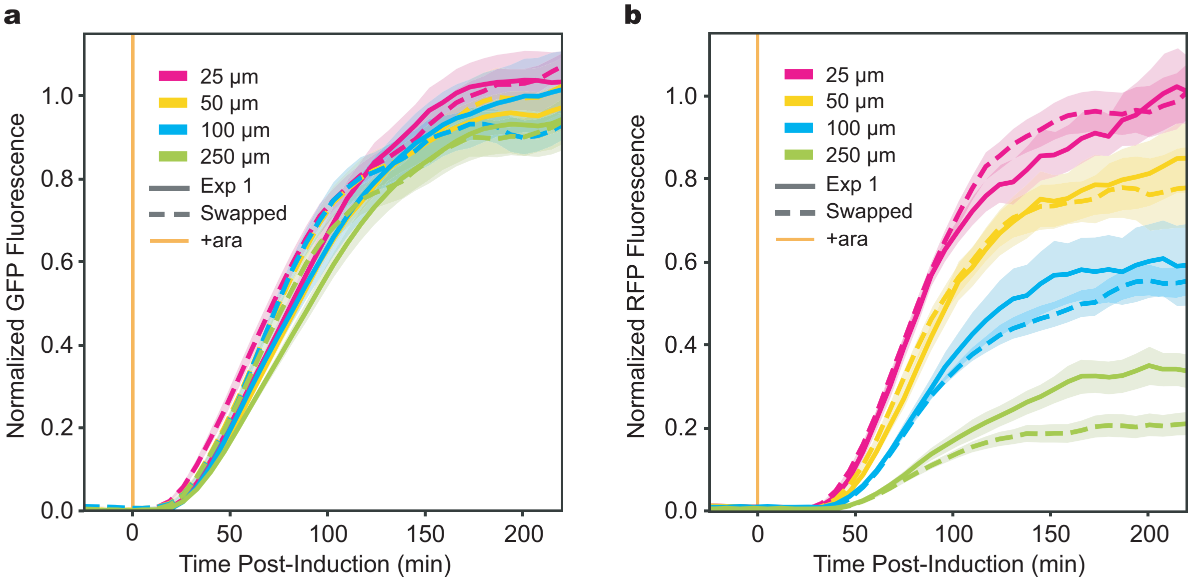

Supplementary Figure 4: Effects of inversion of the positions of the sender and receiver strains on the dynamic response of inter-strain communication. **(a)** GFP fluorescence in sender growth chambers as a function of time for original (dashed line) (Experiment 1, **Table 1**) and inverted (solid line) (Experiment 2, **Table 1**) chamber positions. The vertical orange line indicates the time at which arabinose was introduced. Shaded regions represent one standard deviation from the mean. Fluorescence intensities are normalized between 0 and 1 to aid direct comparison between experiments. A two-sided t-test demonstrated no statistically significant difference between GFP values observed at steady-state (215 min post-induction) across all separation distances ($P > 0.05$) between the two experiments (**Supplementary Fig. 16**). **(b)** RFP fluorescence over time in the receiver growth chambers for original (dashed line) (Experiment 1, **Table 1**) and inverted (solid line) (Experiment 2, **Table 1**) chamber positions. The vertical orange line indicates the time at which arabinose was introduced for both experiments. Shaded regions represent one standard deviation from the mean. Fluorescence intensities are normalized between 0 and 1 to aid direct comparison between experiments. A two-sided t-test demonstrated no statistically significant difference ($P > 0.05$) between RFP values observed at steady-state (215 min post-induction) at 25, 50, and 100 μm distances. A statistically significant difference was observed ($P = 0.01014$) between the two experiments at a separation distance of 250 μm (**Supplementary Fig. 16**). Potential factors contributing to the difference in steady-states in the 250 μm condition include minor differences in initial growth conditions or experimental setup.

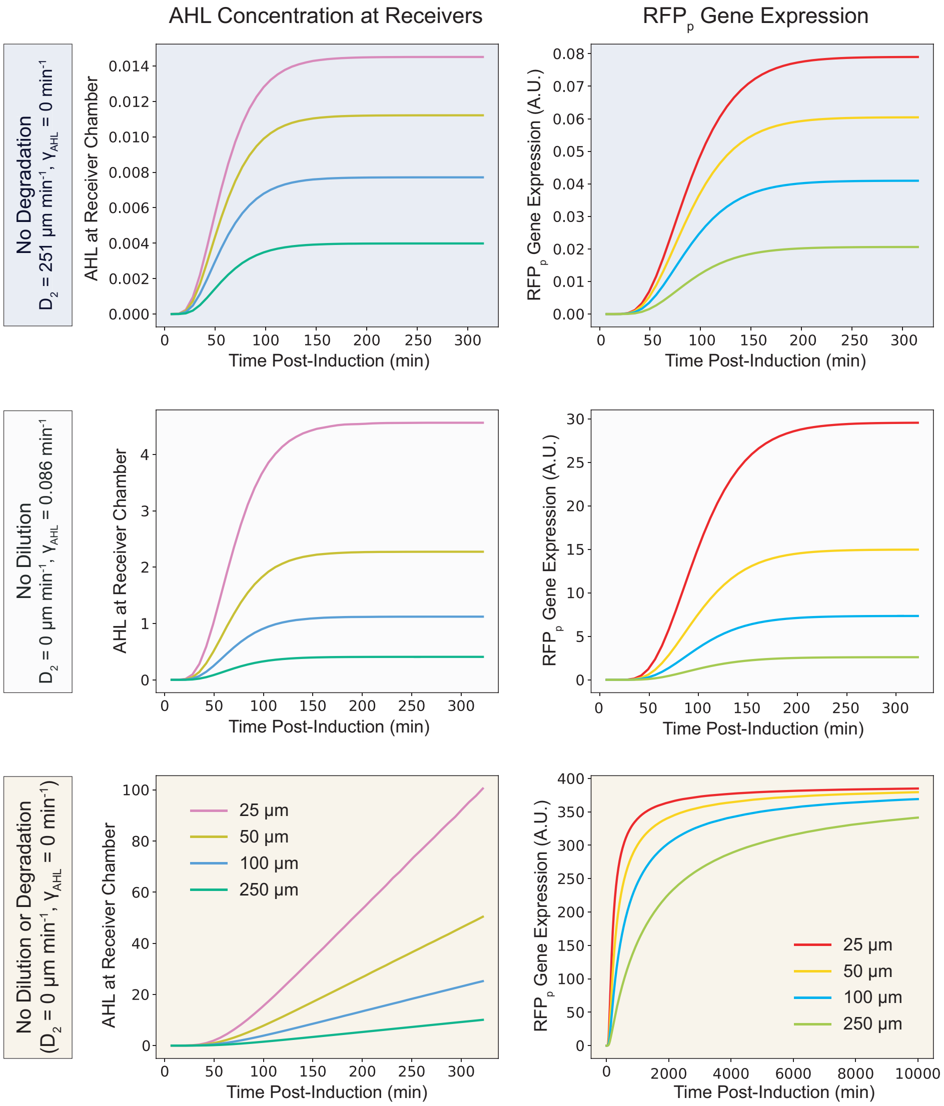

Supplementary Figure 5: Parameter dependence of simulated AHL and RFP_p. Top row: model time-responses of AHL (left column) and RFP_p (right column) where the degradation rate of AHL in the interaction channel is set to zero ($\gamma_{AHL} = 0$). **Middle row:** model time-responses where the diffusion constant of AHL away from the growth chambers into the main channel is set to zero ($D_2 = 0$). **Bottom row:** model time-responses of AHL and RFP_p where $D_2 = 0$ and $\gamma_{AHL} = 0$.

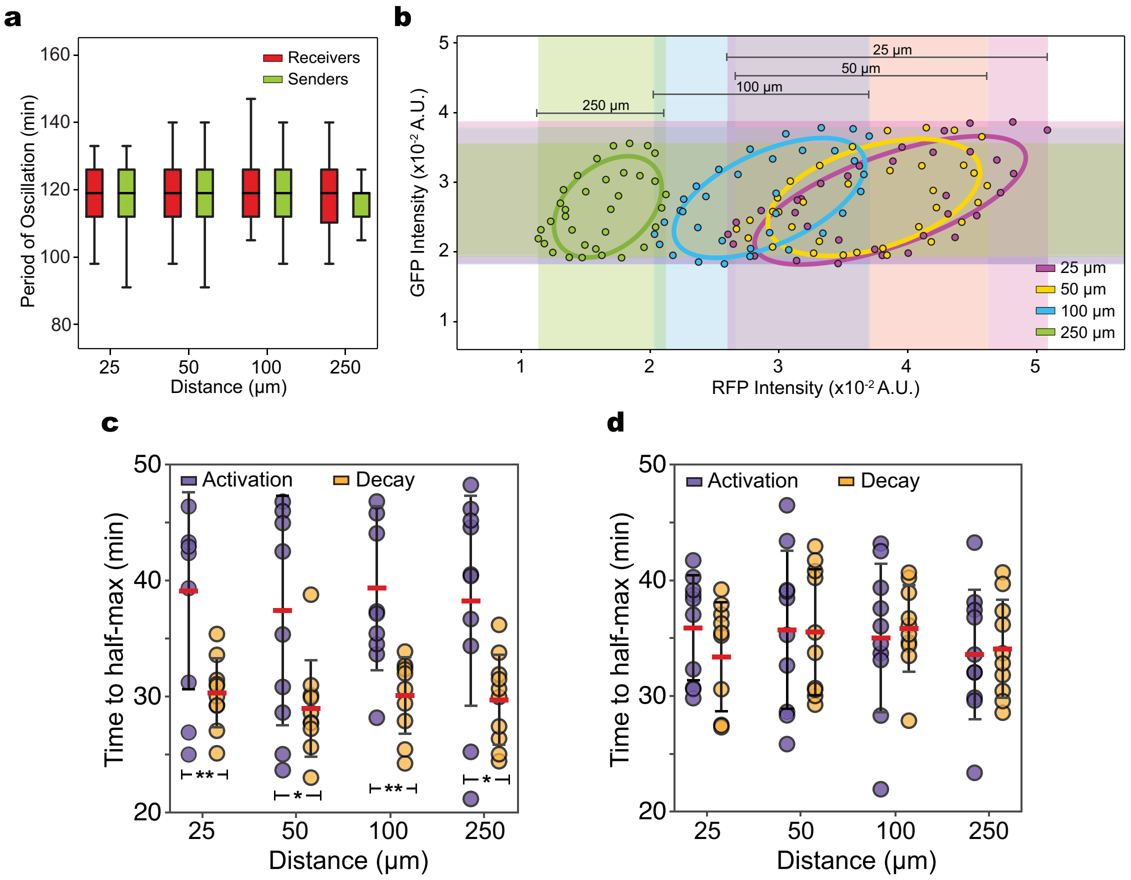

Supplementary Figure 6: Effects of spatial separation on the response of inter-strain communication to a periodic input. **(a)** Box plots of the experimentally measured distribution of oscillatory periods, determined by applying a peak finding algorithm to time-series RFP (receivers) or GFP (senders) data for all cell growth chambers (Experiment 3, **Table 1**). The period was measured as a two hour oscillation (defined as 1 hr exposed to arabinose and 1 hr in the absence of arabinose) between 304 to 1004 min. Horizontal lines within boxes indicate the median and upper and lower box edges represent the upper and lower quartiles, respectively. Upper and lower whiskers represent the 95th and 5th confidence intervals, respectively. A two-sided t-test of oscillatory periods across distance showed no statistical significance (P > 0.05) **(b)** Phase plot of RFP vs. GFP for two oscillatory periods from 580 to 700 min for a forced oscillation experiment with an arabinose period of 2 hr (Experiment 3, **Table 1**). **(c)** Activation and decay response times for GFP (sender strain). Activation response time is defined as the time to reach half-maximum from the minimum to the maximum of each period. The decay response time is defined as the time to reach half-minimum from the maximum to the minimum of each period. Error bars represent one standard deviation from the mean. Horizontal bars with stars indicate a statistically significant difference (P < 0.05) based on a two-sided t-test. One star (*) indicates P < 0.05, two stars (**) indicate $P < 0.01$, and three stars (***) indicate $P < 0.001$. The P-values for the 25, 50, 100 and 250 μm conditions are 0.0064, 0.0229, 0.0015 and 0.0135, respectively. **(d)** Activation and decay response times for RFP (receiver strain). Error bars represent one standard deviation from the mean. A two-sided t-test between activation and decay times showed no statistical significance (P > 0.05)

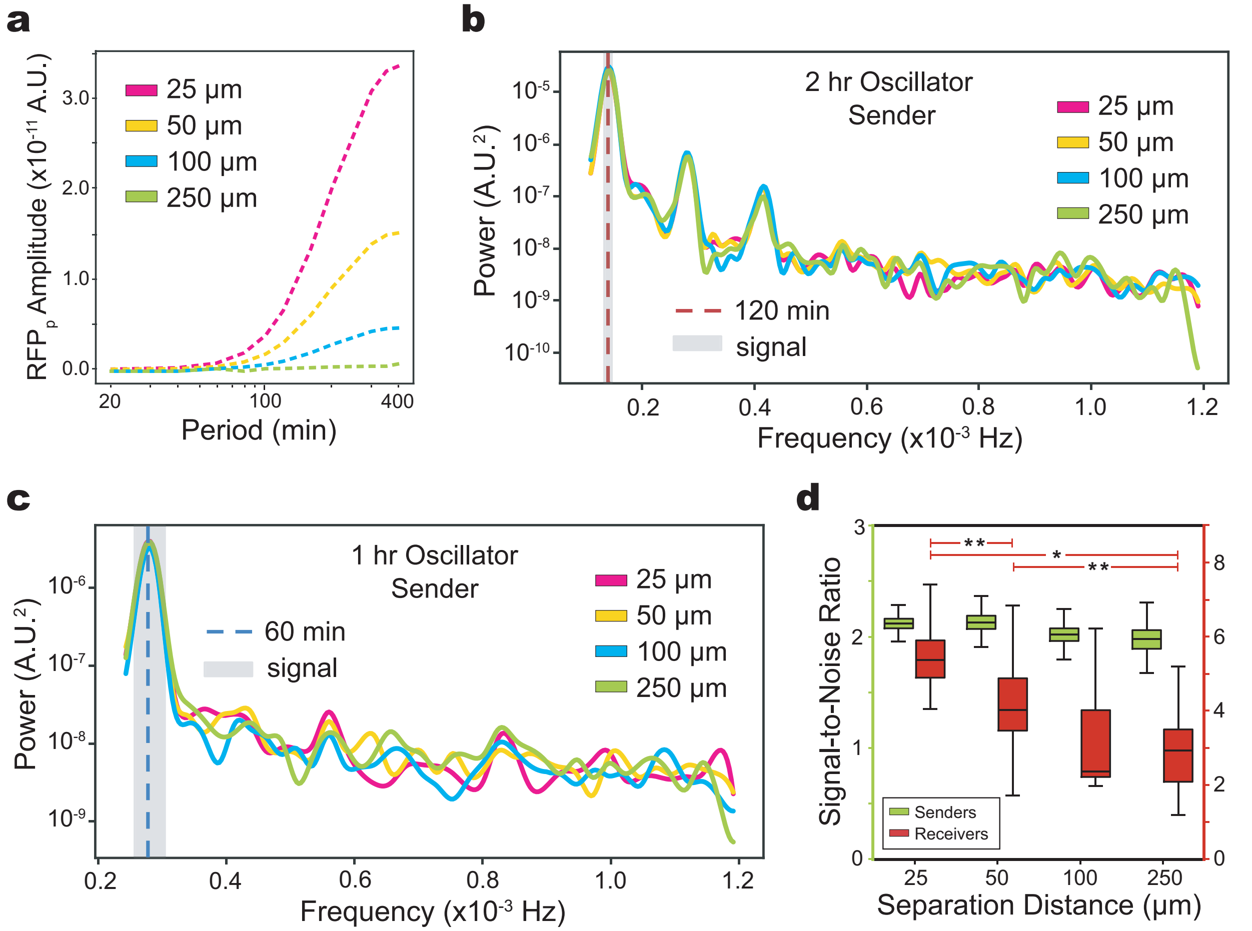

Supplementary Figure 7: Amplitude variation, power spectra, and signal-to-noise ratios for the sender-receiver periodic input experiments. (a) Relationship between the period of an oscillatory arabinose (ara) input and the RFP_p steady-state amplitude in the model for $n_{RFP} = 3$ for different distances from the sender strain. **(b)** Mean power spectra of GFP (sender strain) in response to a periodic input with a period of 2 hr (Experiment 3, **Table 1**). Power spectra were calculated using the Welch method with a Hamming windowing function at steady-state after 395 min (**Methods**). The arabinose frequency is indicated with a dashed red line. The signal bandwidth, denoted by a gray shaded region, is defined as the frequency range corresponding to 115-125 min. **(c)** Mean power spectra of GFP (sender strain) for the forced oscillation experiment input period of 1 hr (Experiment 4, **Table 1**). Power spectra were calculated using the Welch method with a Hamming windowing function at steady-state after 500 min (**Methods**). The arabinose frequency is represented by a dashed red line. The signal bandwidth is represented by a gray shaded region and is defined as the frequency range corresponding to 55-65 min. **(d)** Signal-to-noise (SNR) ratios based on the power spectra of GFP or RFP (receiver strain) across distance for the forced oscillation experiment with an input period of 2 hr (Experiment 3, **Table 1**). Left and right axes denote SNR for GFP and RFP, respectively. Horizontal lines within boxes denote the median and upper and lower edges represent the upper and lower quartiles, respectively. Upper and lower whiskers represent the 95th and 5th confidence intervals, respectively. Horizontal lines with stars (colored green for senders and red for receivers) denote a statistically significant difference ($P < 0.05$) based on one-tailed bootstrap hypothesis testing (**Methods**). One star (*) indicates $P < 0.05$, two stars (**) indicate $P < 0.01$, and three stars (***) indicate $P < 0.001$ (**Supplementary Fig. 16**).

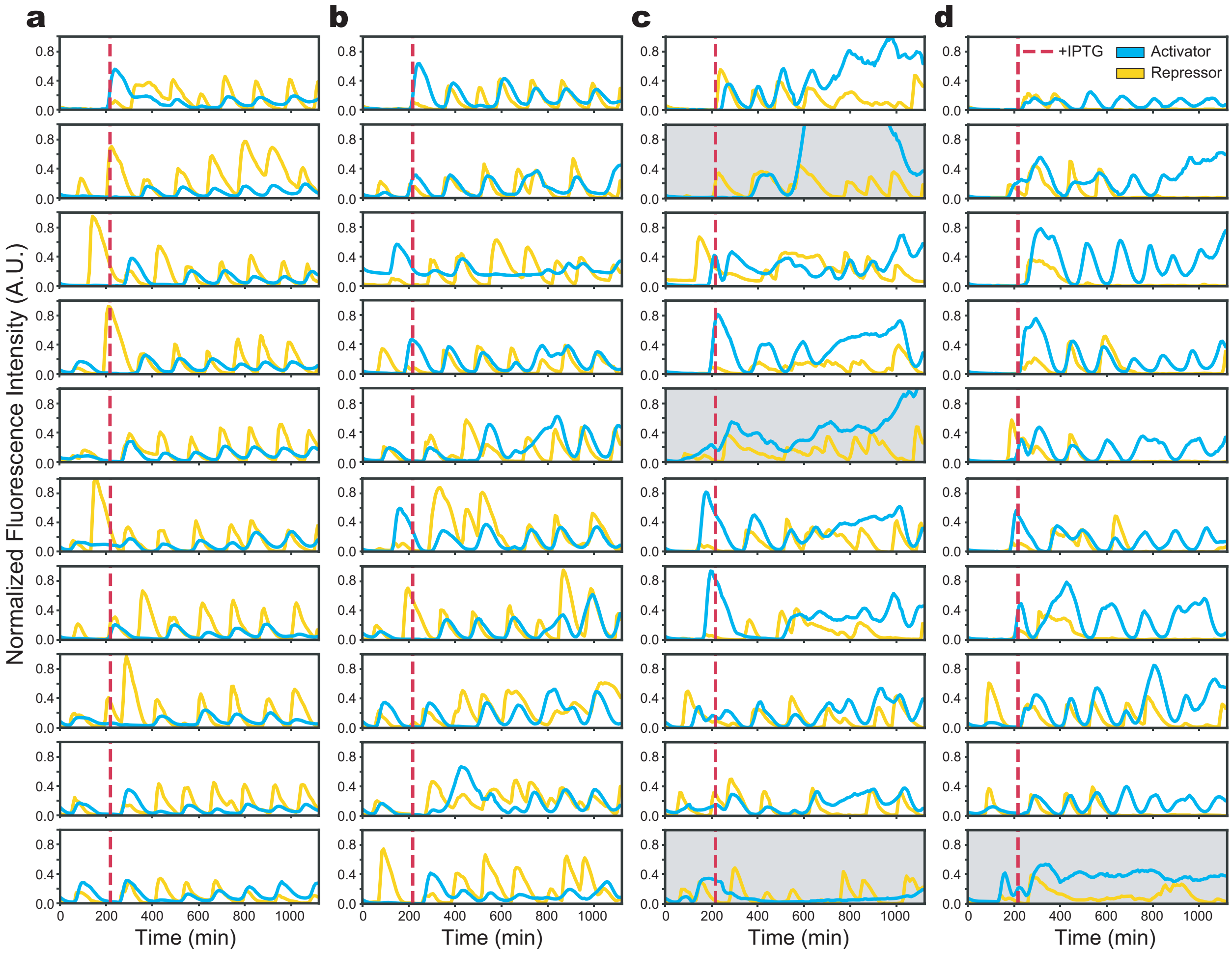

Supplementary Figure 8: Fluorescence intensity over time of dual-feedback oscillators in the presence of 1 mM IPTG (Experiment 5, **Table 1**). **(a)** Normalized CFP (activator) and YFP (repressor) fluorescence intensity as a function of time for a separation distance of 25 μm. The red dashed line indicates the time when IPTG was introduced. For each fluorescence reporter, the fluorescence intensity was normalized between the global minimum and maximum excluding the outliers (gray shaded boxes). **(b)** Normalized CFP (activator) and YFP (repressor) fluorescence intensity as a function of time for a separation distance of 50 μm. **(c)** Normalized CFP (activator) and YFP (repressor) fluorescence intensity as a function of time for a separation distance of 100 μm. **(d)** Normalized CFP (activator) and YFP (repressor) fluorescence intensity as a function of time for a separation distance of 250 μm.

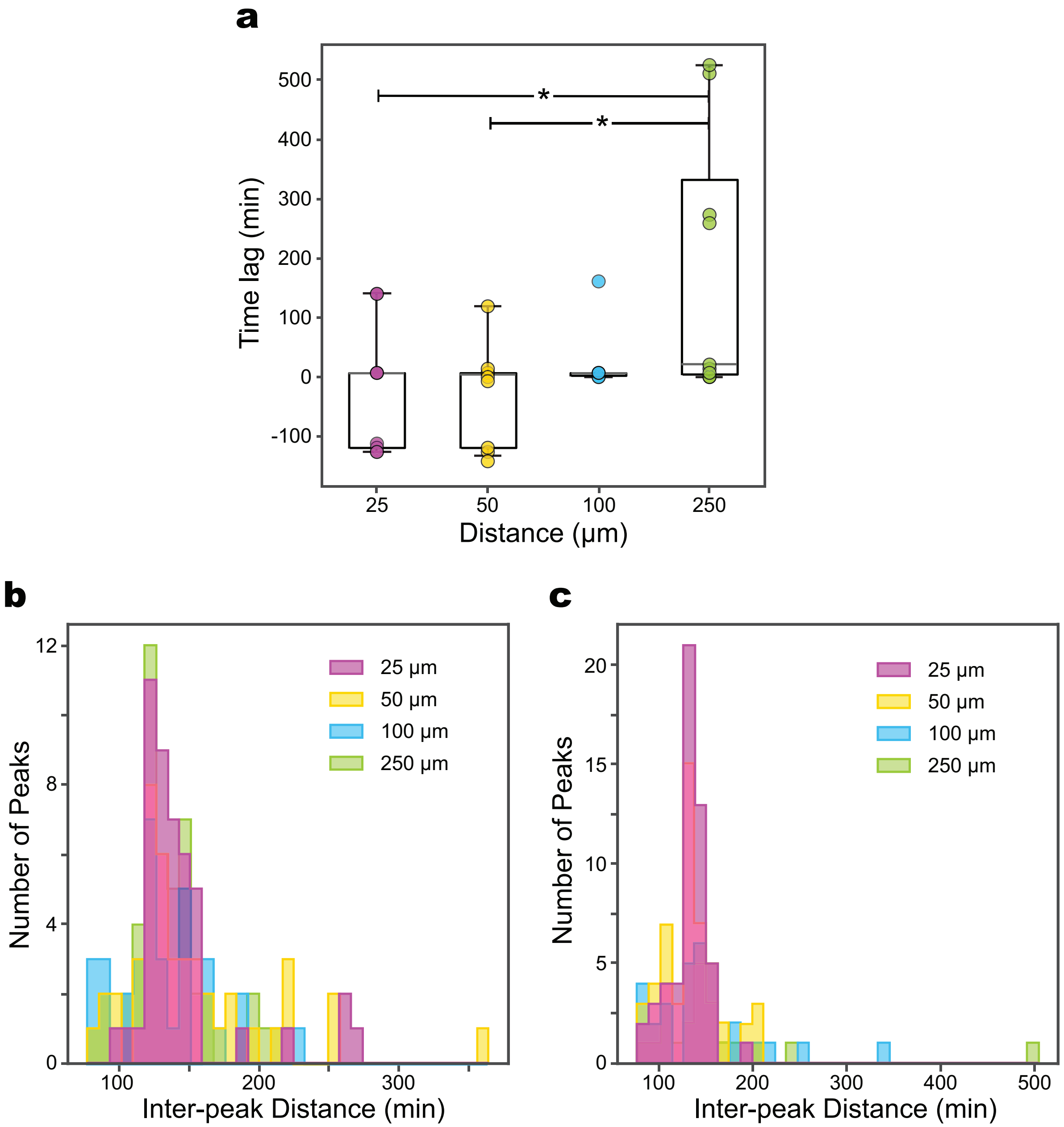

Supplementary Figure 9: Inter-peak distances and time lags of maximum cross-correlation for the dual-feedback oscillator consortium. (Experiment 5, **Table 1**). **(a)** Box plot denoting time lags of maximum cross-correlation between CFP (activator) and YFP (repressor) for paired interaction channels. Horizontal lines within boxes denote the median and upper and lower edges represent the upper and lower quartiles, respectively. Upper and lower whiskers represent the 95th and 5th confidence intervals, respectively. Horizontal bars with stars indicate a statistically significant difference (P < 0.05) based on a two-sided t-test. One star (*) indicates P < 0.05, two stars (**) indicate $P < 0.01$, and three stars (***) indicate $P < 0.001$ (**Supplementary Fig. 16**). **(b)** Histogram of inter-peak distances for CFP (activator). A peak finding algorithm (Python) was used to determine the peaks in the normalized CFP fluorescence intensity as a function of time. CFP was normalized by subtracting a moving mean. The inter-peak distance was computed difference in the times corresponding to each peak for all replicates of a given interaction channel length. **(c)** Histogram of inter-peak distances for YFP (repressor).

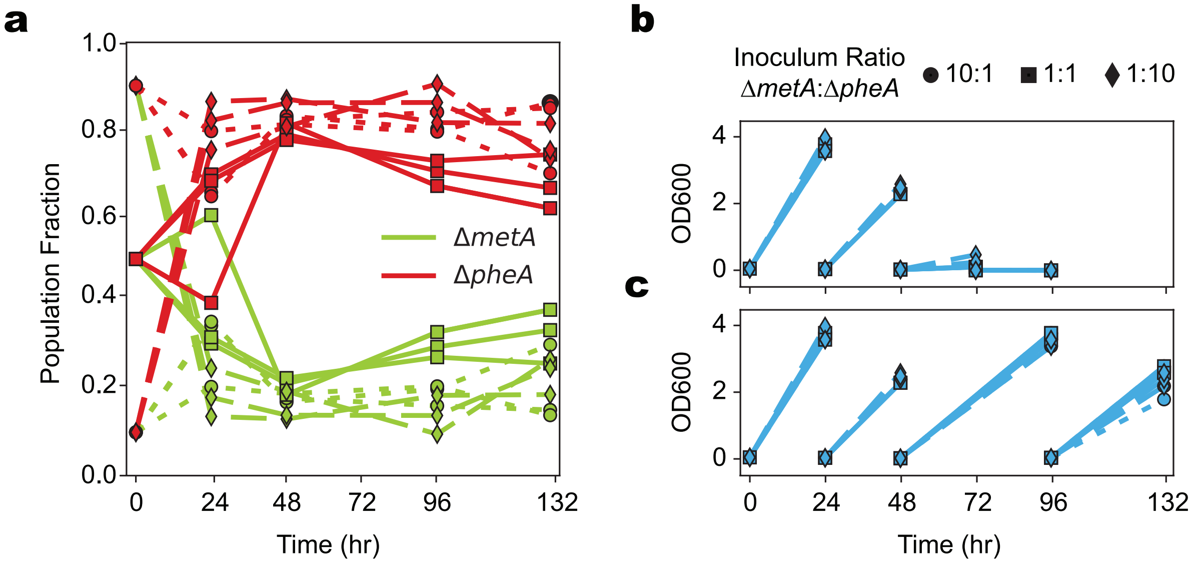

Supplementary Figure 10: Population dynamics of the Δ*metA*-Δ*pheA* consortium in batch culture. **(a)** Fraction of each strain as a function of time. Marker and line styles correspond to different initial ratios of Δ*metA* to Δ*pheA*. Circles and dotted lines represent 10:1, squares and solid lines represent 1:1, and diamonds and dashed lines represent 1:10. **(b)** OD600 measurements as a function of time for a passaging period maintained at 24 hours. **(c)** OD600 measurements as a function of time for a second condition where the incubation period after the second passage was extended to 48 hr. The number of cells counted for each condition ranged between 250-3960.

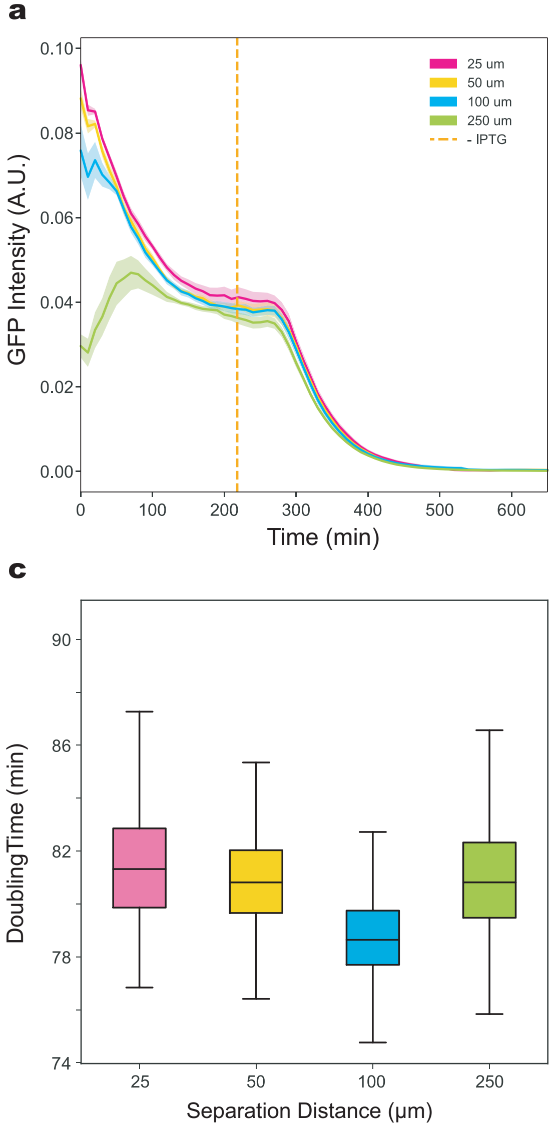

Supplementary Figure 11: Fluorescence as a function of time and doubling time distributions for the +M +F condition. (Experiment 6, **Table 1**). **(a)** GFP fluorescence in the $\Delta metA$ strain as a function of time in media supplemented with all amino acids. The dashed line indicates the time of the media switch from media containing IPTG to media lacking IPTG. Solid lines and shaded regions represent the mean and one standard deviation from the mean, respectively. **(b)** RFP fluorescence in the $\Delta pheA$ strain over time in media supplemented with all amino acids. The dashed line represents the media switch for removal of IPTG. **(c)** Box plot of cell doubling times for $\Delta metA$ at each interaction channel length. Horizontal lines within boxes depict the median and upper and lower edges represent the upper and lower quartiles, respectively. Upper and lower whiskers represent the 95th and 5th confidence intervals, respectively. Differences between the distributions represented in the box plot were not statistically significant based on one-tailed bootstrap hypothesis testing (**Supplementary Fig. 17**). **(d)** Box plot of the $\Delta pheA$ doubling times at each interaction channel length. Horizontal lines within boxes denote the median and upper and lower edges represent the upper and lower quartiles, respectively. Upper and lower whiskers represent the 95th and 5th confidence intervals, respectively. Differences between the distributions represented in the box plot were not statistically significant based on one-tailed bootstrap hypothesis testing (**Supplementary Fig. 17**).

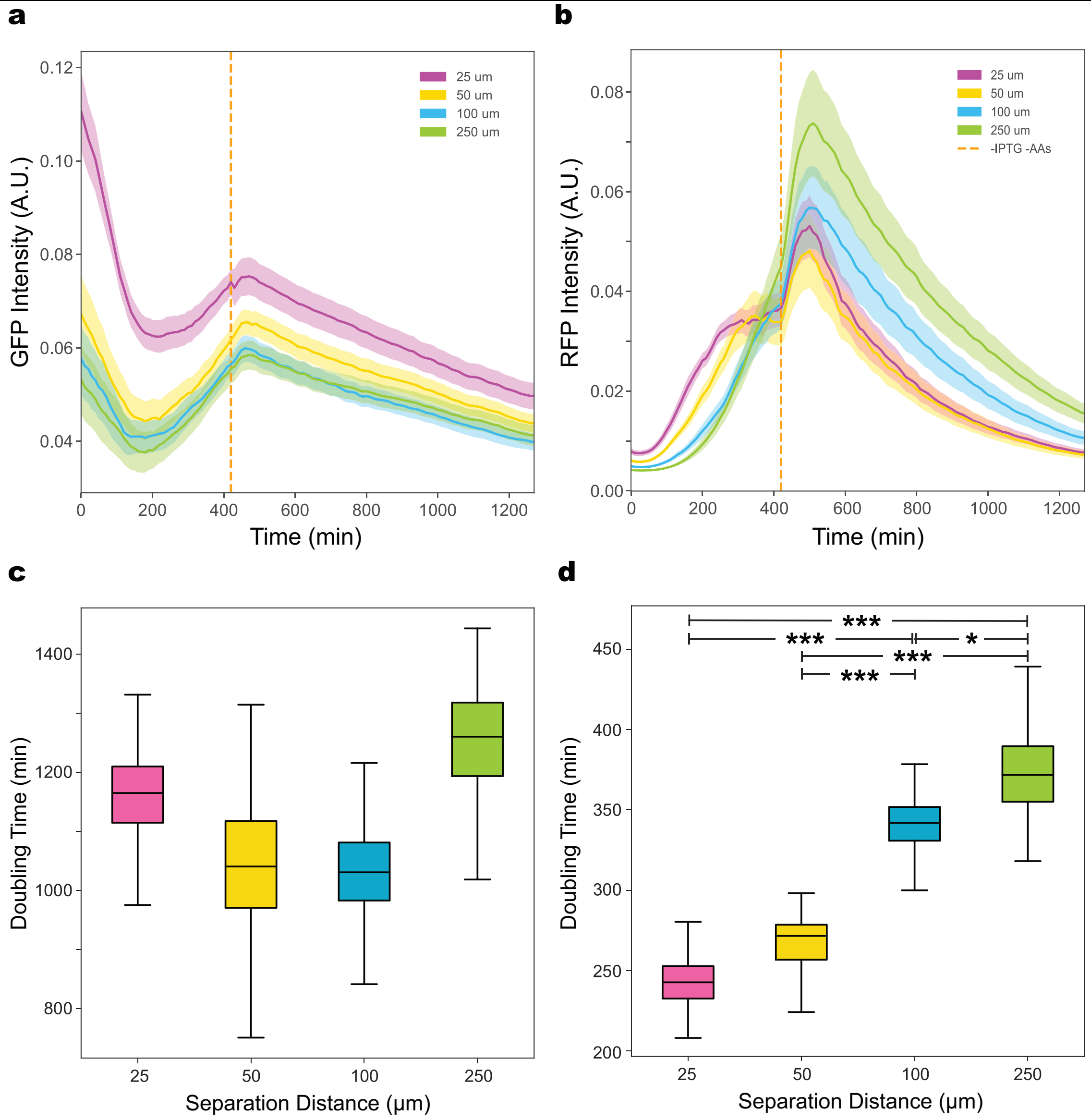

Supplementary Figure 12: Fluorescence as a function of time and doubling time distributions in the absence of F and M. (Experiment 7, **Table 1**). **(a)** GFP fluorescence over time in the $\Delta metA$ strain at different distances from the partner strain ($\Delta pheA$). The dashed line represents the time when the media was switched from pre-culture to test conditions. Solid lines and shaded regions represent the mean and one standard deviation from the mean, respectively. **(b)** RFP fluorescence over time in the $\Delta pheA$ strain at different distances from the partner strain ($\Delta metA$). **(c)** Box plot of the cell doubling times for the $\Delta metA$ strain. Horizontal lines within boxes denote the median and upper and lower edges represent the upper and lower quartiles, respectively. Upper and lower whiskers represent the 95th and 5th confidence intervals, respectively. Differences between the distributions represented in the box plot were not statistically significant based on one-tailed bootstrap hypothesis testing (**Supplementary Fig. 17**). **(d)** Box plot of the doubling times for $\Delta pheA$ strain. Horizontal lines within boxes denote the median and upper and lower edges represent the upper and lower quartiles, respectively. Upper and lower whiskers represent the 95th and 5th confidence intervals, respectively. Horizontal bars with stars indicate a statistically significant difference (P $<$ 0.05) based on one-tailed bootstrap hypothesis testing. One star (*) indicates P $<$ 0.05, two stars (**) indicate $P < 0.01$, and three stars (***) indicate $P < 0.001$ (**Supplementary Fig. 17**).

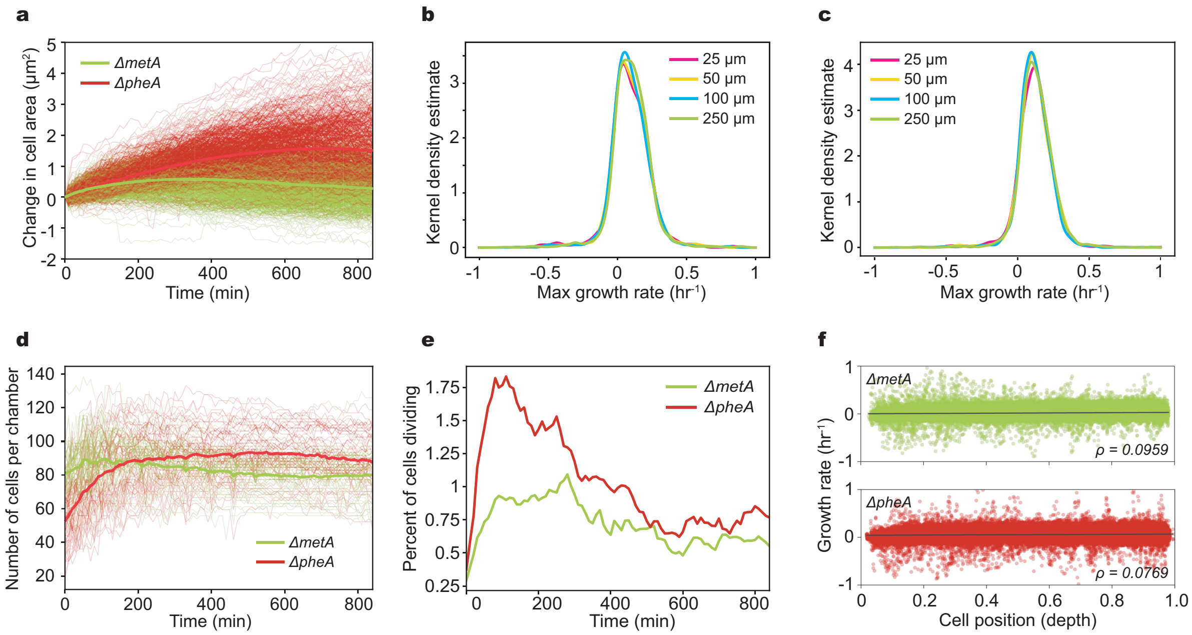

Supplementary Figure 13: Quantification of single-cell growth in the mixed auxotroph condition (Experiment 8, **Table 1**). **(a)** Change in cell area as a function of time for $\Delta metA$ (n = 937) and $\Delta pheA$ (n = 662). The change in cell area is defined as the cumulative change in cross-sectional area of each cell from its initial value at the time of the media switch. Thin and bold lines represent individual cells and the strain averages, respectively. **(b)** Kernel density estimate distribution of $\Delta metA$ single cell growth rates over 14 hr post media switch in the mixed auxotroph experiment for each interaction channel length. **(c)** Kernel density estimate distribution of $\Delta pheA$ single cell growth rates over 14 hr post media switch in the mixed auxotroph experiment for each interaction channel length. **(d)** Total number of $\Delta metA$ and $\Delta pheA$ cells in each growth chamber as a function of time. Bold lines represent the average number of cells for each strain across all growth chambers. **(e)** Percent of $\Delta metA$ or $\Delta pheA$ cells dividing as a function of time. The fraction of dividing cells was computed using a window spanning 60 min starting at each time point, which was then divided by the average number of $\Delta metA$ or $\Delta pheA$ cells in the window. **(f)** Scatter plot of the correlation between the growth rate and depth position of individual $\Delta metA$ (n=165408) and $\Delta pheA$ (n=193970) cells in a mixed community (Experiment 8, **Table 1**). Positions zero and one represent the outermost and deepest regions of the growth chamber, respectively, relative to the main channels. Solid bold lines show simple linear regression between maximum growth rate and chamber position. The Pearson correlation coefficient is 0.0959 ($P < 0.001$) for $\Delta metA$ and 0.0769 ($P < 0.001$) for $\Delta pheA$.

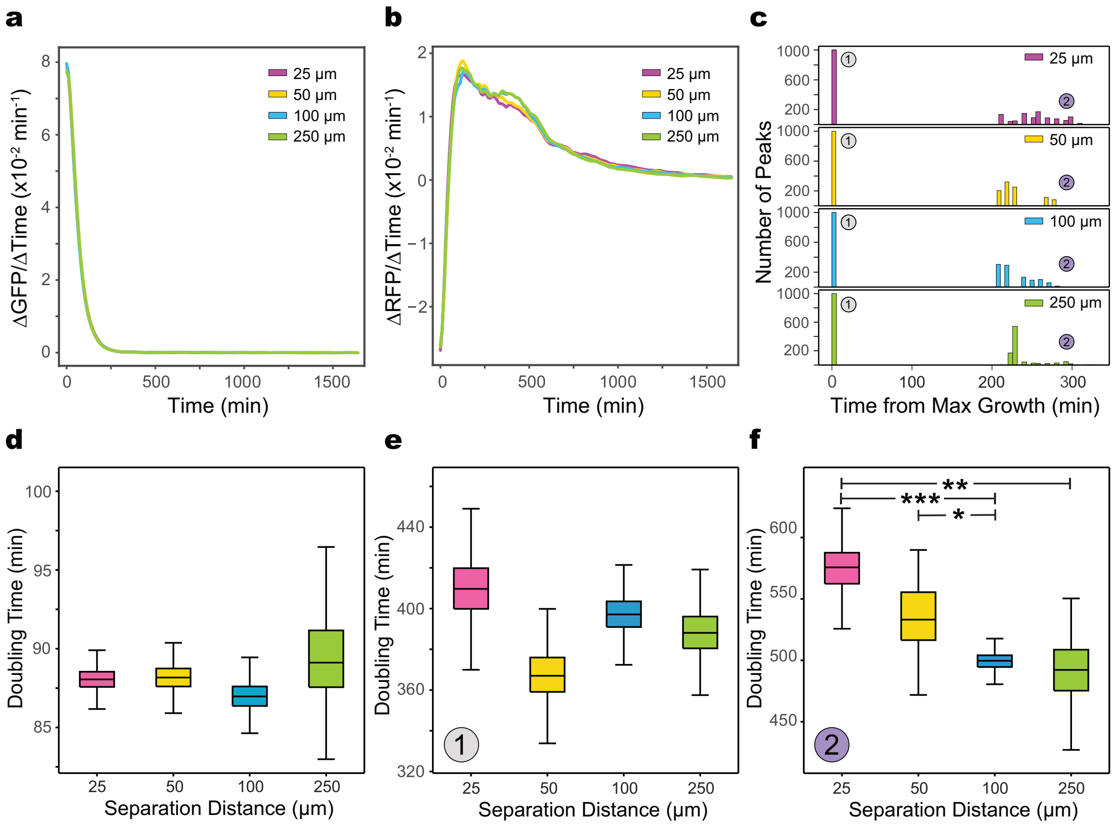

Supplementary Figure 14: Characterization of population-level growth rates of amino acid auxotrophs in the presence of M and absence of F (Experiment 11, **Table 1**). **(a)** Rate of change in GFP fluorescence of Δ*metA* as a function of time. Solid lines and shaded regions represent the mean and one standard deviation from the mean, respectively. **(b)** Rate of change of RFP fluorescence of Δ*pheA* over time. **(c)** Histogram of the number of peaks identified in the rate of change of RFP fluorescence over time for the 1000 bootstrapped curves using a peak finding algorithm (**Methods**). The distributions were normalized to the time of maximum growth rate. **(d)** Box plot of cell doubling times for the Δ*metA* strain at different distances from the Δ*pheA* strain. Horizontal lines within boxes depict the median and upper and lower edges represent upper and lower quartiles, respectively. Upper and lower whiskers represent the 95th and 5th confidence intervals, respectively. Differences between the distributions represented in the box plot were not statistically significant based on one-tailed bootstrap hypothesis testing (**Supplementary Fig. 17**). **(e)** Box plot of cell doubling times for first growth phase corresponding to the maximum growth rate of the Δ*pheA* strain. Horizontal lines within boxes denote the median and upper and lower edges represent the upper and lower quartiles, respectively. Upper and lower whiskers represent the 95th and 5th confidence intervals, respectively. Differences between the distributions represented in the box plot were not statistically significant based on one-tailed bootstrap hypothesis testing (**Supplementary Fig. 17**). **(f)** Box plot of the cell doubling times for second growth phase of the Δ*pheA* strain. Horizontal lines within boxes denote the median and upper and lower edges represent the upper and lower quartiles, respectively. Upper and lower whiskers represent the 95th and 5th confidence intervals, respectively. The horizontal bars denote a statistical significant difference (P < 0.05) based on one-tailed bootstrap hypothesis testing. One star (*) indicates P < 0.05, two stars (**) indicate $P < 0.01$, and three stars (***) indicate $P < 0.001$ (**Supplementary Fig. 17**).

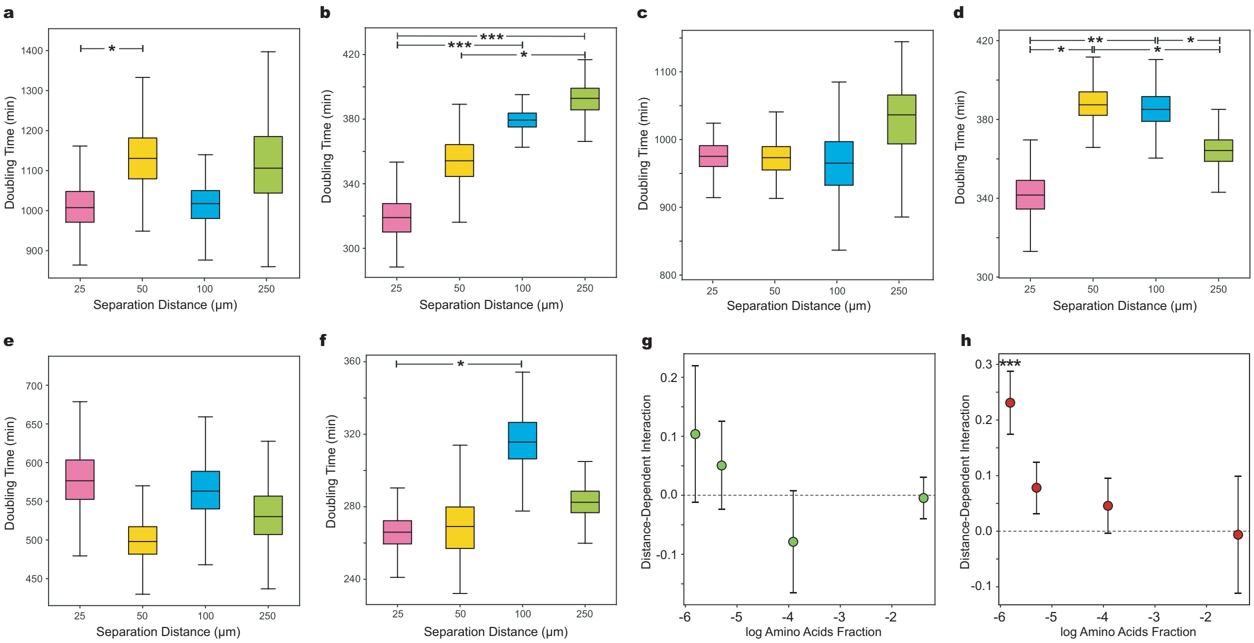

Supplementary Figure 15: Population-level growth rates of amino-acid auxotrophs across different concentrations of supplemented amino acids. For all box plots in the figure, horizontal lines within boxes denote the median and upper and lower edges represent the upper and lower quartiles, respectively. Upper and lower whiskers represent the 95th and 5th confidence intervals, respectively. For all panels displaying box plots, the horizontal bars denote a statistically significance difference (P $<$ 0.05) based on one-tailed bootstrap hypothesis testing (**Supplementary Fig. 17**). **(a)** Box plot of the doubling times for the $\Delta metA$ strain in Experiment 12 (**Table 1**). **(b)** Box plot of the cell doubling times for the $\Delta pheA$ strain for Experiment 12. **(c)** Box plot of the cell doubling times for the $\Delta metA$ strain in Experiment 13. **(d)** Box plot of the cell doubling times for the $\Delta pheA$ strain in Experiment 13. **(e)** Box plot of the cell doubling times for the $\Delta metA$ strain in Experiment 14. **(f)** Box plot of the cell doubling times for the $\Delta pheA$ strain in Experiment 14. **(g)** Relationship between the fraction of all amino acids and the sensitivity of growth of $\Delta metA$ to spatial separation from $\Delta pheA$ (Experiments 6, 12-14 **Table 1**). The distance-dependent interaction value is defined as the ratio of the minimum doubling time of each strain in the 25 μm to 250 μm condition ($D_{25}D_{250}^{-1}$) minus 1. Error bars represent one standard deviation from the mean interaction value. A positive interaction was greater than zero and a negative interaction was less than zero. No data points showed statistically significant differences (P $<$ 0.05) between D_{25} and D_{250}, using a two-sided t-test. **(h)** Relationship between the fraction of all amino acids and the sensitivity of growth of $\Delta pheA$ (interaction strength) to spatial separation from $\Delta metA$ (Experiments 6, 12-14 **Table 1**). Error bars represent one standard deviation from the mean interaction value. Three stars (***) indicate P = 0.00040 for the difference in D_{25} and D_{250} using a two-sided t-test. No other data points showed statistically significant differences (P $<$ 0.05) between D_{25} and D_{250}.

Supplementary Fig. 4a,b - Experiments 1-2 (inverted positions)

sender

25	50	100	250
0.402259	0.608653	0.222456	0.936418

receiver

25	50	100	250
0.456543	0.735272	0.490299	0.01014

Supplementary Fig. 7d - Experiment 3 (SNR)

sender

	50	100	250
25	0.4142	0.4865	0.4724
50		0.486	0.496
100			0.4009

receiver

	50	100	250
25	0.0084	0.0946	0.0386
50		0.1281	0.0038
100			0.3634

Fig. 3i - Experiment 4 (SNR)

sender

	50	100	250
25	0.3011	0.5635	0.6508
50		0.2185	0.4167
100			0.5957

receiver

	50	100	250
25	0.041243	0.000008	0.000063
50		0.006158	0.007676
100			0.111161

Fig. 4c - Experiment 5 (amplitudes)

activator

	50	100	250
25	0.00027	0.000882	0.0003684
50		0.989946	0.2593051
100			0.3396577

repressor

	50	100	250
25	0.490548	0.00281	0.006595
50		0.01196	0.026273
100			0.82516

Fig. 4d - Experiment 5 (number of peaks)

activator

	50	100	250
25	0.530488	0.082501	0.0121141
50		0.074239	0.0180749
100			0.7831872

repressor

	50	100	250
25	0.482493	0.116482	7.36E-06
50		0.221686	1.12E-05
100			0.003286

Fig. 4e - Experiment 5 (maximum correlation)

	50	100	250
25	0.3311	0.0868	0.0002
50		0.2526	0.0003
100			0.009

Supplementary Fig. 9a - Experiment 5 (time lag)

	50	100	250
25	0.893	0.3204	0.0216
50		0.1789	0.0149
100			0.1008

Supplementary Figure 16: P-values for quorum-sensing experiments. Two-sided t-tests or bootstrap hypothesis testing (**Methods**) were used to compute the P-values for Experiments 1-5 (**Table 1**). Highlighted values indicate statistically significant differences ($P < 0.05$).

Fig. 5b, Supplementary Fig. 11c - Experiment 6 (+M +F)

ΔmetA	50	100	250
25	0.968962	0.222213	0.6677918
50		0.175572	0.6213711
100			0.4308596

Fig. 5b, Supplementary Fig. 12c - Experiment 7 - Auxotroph (-M -

ΔmetA	50	100	250
25	0.070324	0.131259	0.288765
50		0.750636	0.413298
100			0.622076

Fig. 5c, Supplementary Fig. 11d - Experiment 6 (+M +F)

ΔpheA	50	100	250
25	0.108647	0.801381	0.4505158
50		0.191799	0.1860261
100			0.6497227

Fig. 5c, Supplementary Fig. 12d - Experiment 7 (-M -F)

ΔpheA	50	100	250
25	0.364665	5.57E-06	6.29E-06
50		1.05E-05	1.49E-05
100			0.027184

Fig. 5c, inset - Experiment 7 (-M -F time lag)

ΔpheA	50	100	250
25	0.3355	0.0432	0.0494
50		0.0682	0.1185
100			0.0935

Fig. 5f, Supplementary Fig. 14d - Experiment 11 (+M -F)

ΔmetA	50	100	250
25	0.896383	0.424424	0.774661
50		0.394931	0.817224
100			0.535158

Fig. 5f, Supplementary Fig. 14e - Experiment 11 (+M -F)

ΔpheA GP1	50	100	250
25	0.489943	0.55232	0.8350058
50		0.138334	0.5632902
100			0.3307124

Fig. 5f, Supplementary Fig. 14f - Experiment 11 (+M -F)

ΔpheA GP2	50	100	250
25	0.182705	0.000366	0.004516
50		0.020797	0.089901
100			0.624952

Supplementary Fig. 15a - Experiment 12 (0.003X AAs)

ΔmetA	50	100	250
25	0.037385	0.641391	0.5535304
50		0.098395	0.2834969
100			0.8037953

Supplementary Fig. 15b - Experiment 12 (0.003X AAs)

ΔpheA	50	100	250
25	0.064692	0.000759	0.000389
50		0.120851	0.043729
100			0.287352

Supplementary Fig. 15c - Experiment 13 (0.005X AAs)

ΔmetA	50	100	250
25	0.247728	0.754728	0.0822178
50		0.505827	0.2253888
100			0.1477334

Supplementary Fig. 15d - Experiment 13 (0.005X AAs)

ΔpheA	50	100	250
25	0.011602	0.009112	0.496241
50		0.673519	0.029181
100			0.022791

Supplementary Fig. 15e - Experiment 14 (0.02X AAs)

ΔmetA	50	100	250
25	0.05376	0.943291	0.6221304
50		0.057607	0.1632572
100			0.6682308

Supplementary Fig. 15f - Experiment 14 (0.02X AAs)

ΔpheA	50	100	250
25	0.58644	0.040782	0.18212
50		0.288083	0.691512
100			0.305052

Fig. 5g - M and F assays

$M_{\Delta pheA}$	0.01X	0.025X	0.05X	1.0X
0X	0.154073	0.162219	0.1289866	0.1045635
0.01X		0.697201	0.2347936	0.0101755
0.025X			0.377097	0.1538273
0.05X				0.110678

$F_{\Delta metA}$	0.01X	0.025X	0.05X	1.0X
0X	0.118566	0.051864	0.031624	0.010126
0.01X		0.523527	0.119889	0.01876512
0.025X			0.04259	0.00445829
0.05X				0.00081317

Supplementary Figure 17: P-values for auxotroph experiments. Two-sided t-tests or bootstrap hypothesis testing (**Methods**) were used to compute the P-values for auxotroph Experiments 6-7, 11-14 and M and F amino acid measurements (**Table 1**). Highlighted values indicate statistically significant differences ($P < 0.05$).
