# Supplementary material for: Investigating the dynamics of microbial consortia in spatially structured environments
Source: Nat Commun. 2020 May 15;11:2418. doi: 10.1038/s41467-020-16200-0 (PMC7228966; doi:10.1038/s41467-020-16200-0)
Supplement: Supplementary file 7 — Reporting Summary [file 41467_2020_16200_MOESM7_ESM.pdf]

nature research

Corresponding author(s): Ophelia S. Venturelli

Last updated by author(s): 04/11/2020

Reporting Summary

Nature Research wishes to improve the reproducibility of the work that we publish. This form provides structure for consistency and transparency in reporting. For further information on Nature Research policies, see Authors & Referees and the Editorial Policy Checklist .

Statistics

For all statistical analyses, confirm that the following items are present in the figure legend, table legend, main text, or Methods section.

n/a	Confirmed	
☐	☒	The exact sample size (*n*) for each experimental group/condition, given as a discrete number and unit of measurement
☐	☒	A statement on whether measurements were taken from distinct samples or whether the same sample was measured repeatedly
☐	☒	The statistical test(s) used AND whether they are one- or two-sided<br>*Only common tests should be described solely by name; describe more complex techniques in the Methods section.*
☒	☐	A description of all covariates tested
☐	☒	A description of any assumptions or corrections, such as tests of normality and adjustment for multiple comparisons
☐	☒	A full description of the statistical parameters including central tendency (e.g. means) or other basic estimates (e.g. regression coefficient) AND variation (e.g. standard deviation) or associated estimates of uncertainty (e.g. confidence intervals)
☐	☒	For null hypothesis testing, the test statistic (e.g. *F*, *t*, *r*) with confidence intervals, effect sizes, degrees of freedom and *P* value noted<br>*Give P values as exact values whenever suitable.*
☒	☐	For Bayesian analysis, information on the choice of priors and Markov chain Monte Carlo settings
☒	☐	For hierarchical and complex designs, identification of the appropriate level for tests and full reporting of outcomes
☐	☒	Estimates of effect sizes (e.g. Cohen's *d*, Pearson's *r*), indicating how they were calculated

Our web collection on statistics for biologists contains articles on many of the points above.

Software and code

Policy information about availability of computer code

Data collection	NIS Elements AR 4.51.00 was used for image collection by the TI-Eclipse inverted microscope (Nikon). The mPG101 Exposure Wizard was used to program the mPG101 laser pattern generator (Heidelberg Instruments).
Data analysis	For population-level analysis, FIJI 2.0.0-rc-65 was used to resize microscopy images and create binary masks for segmentation. DeepCell 2.0 image segmentation software was used to train neural networks to identify the locations of cell growth chambers within microscopy images. Custom code (Python version 2.7) was used to determine the average fluorescence intensity within each growth chamber over time, perform data analysis and visualization, peak finding, and model function fitting. MATLAB 2017b was used for computational modeling, including parameter optimization and simulations. For experiments that did not use DeepCell for segmentation, the raw data from NIS Elements was processed with a custom Python script that corrected translational drift and parsed the file into a set of multipage tiff files. FIJI was used to crop individual growth chambers from each field of view. For single cell segmentation, we trained a convolution neural network using Python with the Keras API and Tensorflow as the back-end. Ground truth segmentations for training were acquired with Ilastik. For cell tracking we trained a second convolution neural network using the same environment. Training data was curated with custom code in MATLAB. Lineage reconstruction was performed in Python with custom scripts that for each cell recorded strain, cell area, growth rate, daughters, and fluorescence. Single-cell data analysis and visualization was performed in Python. Modeling code is available at 10.5281/zenodo.3748013.

For manuscripts utilizing custom algorithms or software that are central to the research but not yet described in published literature, software must be made available to editors/reviewers. We strongly encourage code deposition in a community repository (e.g. GitHub). See the Nature Research guidelines for submitting code & software for further information.

Data

Policy information about availability of data

All manuscripts must include a data availability statement. This statement should provide the following information, where applicable:

- Accession codes, unique identifiers, or web links for publicly available datasets
- A list of figures that have associated raw data
- A description of any restrictions on data availability

Raw data is available at 10.5281/zenodo.3748013.

Field-specific reporting

Please select the one below that is the best fit for your research. If you are not sure, read the appropriate sections before making your selection.

[x] Life sciences [ ] Behavioural & social sciences [ ] Ecological, evolutionary & environmental sciences

For a reference copy of the document with all sections, see nature.com/documents/nr-reporting-summary-flat.pdf

Life sciences study design

All studies must disclose on these points even when the disclosure is negative.

Sample size	We selected 10 growth chambers for each strain for each interaction channel length because this provided a sufficient number of biological replicates to analyze the variability in growth and gene expression, while allowing high temporal resolution for microscopy imaging. For the mixed and monoculture auxotroph experiments, we used all 80 growth chambers available in MISTiC. For the conditioned media experiment, we used a sample size of 3 for each condition under each strain because this allowed us to evaluate the biological variability of the growth and community composition while enabling a manageable experiment.
Data exclusions	Specific criteria were used to eliminate outlier growth chambers from the data sets including (1) infrequent pressure fluctuations leading to loss of cells from the growth chambers, (2) cell growth breaching the interaction channels due to barrier instability from cell loading, (3) collapsed interaction channels due to problems with device bonding, (4) vacant or unfilled growth chambers at the time of induction, (5) cell aggregation near the growth chamber with the potential to alter diffusion, (6) the presence of long, sick cells that perturb observations of other cells within the growth chamber, or (7) loss of optical focus.
Replication	All experiments in the paper have been replicated at least two independent times with the exception of Experiment 10, a control auxotroph monoculture experiment and Experiments 12-14, which were performed to study a trend across different concentrations of amino acids. Replication of the experiments in the paper reproduced the trends that are shown in the figures of the paper. In addition, batch co-cultures of the auxotroph strains were not repeated using identical methods. The batch culture experiment with the amino acid auxotroph community (Figure S9) was repeated twice but with different experimental protocols.
Randomization	A random subset of cells were seeded into growth chambers from a single bacterial culture. The location of strains in the device were inverted to detect any possible convective biases in the device, with none observed.
Blinding	Blinding was not relevant to the study since we were studying the effects of inter-strain communication and amino acid cross-feeding under specific spatial and environmental conditions. As such, the experimental conditions were known to the investigators throughout the study.

Reporting for specific materials, systems and methods

We require information from authors about some types of materials, experimental systems and methods used in many studies. Here, indicate whether each material, system or method listed is relevant to your study. If you are not sure if a list item applies to your research, read the appropriate section before selecting a response.

Materials & experimental systems

n/a	Involved in the study	
[x]	[ ]	Antibodies
[x]	[ ]	Eukaryotic cell lines
[x]	[ ]	Palaeontology
[x]	[ ]	Animals and other organisms
[x]	[ ]	Human research participants
[x]	[ ]	Clinical data

Methods

n/a	Involved in the study	
[x]	[ ]	ChIP-seq
[x]	[ ]	Flow cytometry
[x]	[ ]	MRI-based neuroimaging
